# Cloning and Protein Expression of *eccB_5_* Gene in ESX-5 System from *Mycobacterium tuberculosis*

**DOI:** 10.1089/biores.2019.0019

**Published:** 2020-03-31

**Authors:** Siti Kurniawati, Ni Made Mertaniasih, Manabu Ato, Toshiki Tamura, Soedarsono Soedarsono, Aulanni'am Aulanni'am, Shigetarou Mori, Yumi Maeda, Tetsu Mukai

**Affiliations:** ^1^ Faculty of Medicine, Universitas Airlangga, Surabaya, Indonesia.; ^2^ Department of Clinical Microbiology, Faculty of Medicine, Universitas Airlangga, Surabaya, Indonesia.; ^3^ Department of Mycobacteriology, Leprosy Research Center, National Institute of Infectious Diseases, Tokyo, Japan.; ^4^ Department of Pulmonology and Respiratory Medicine, Faculty of Medicine, Universitas Airlangga, Surabaya, Indonesia.; ^5^ Department of Biochemistry, Faculty of Veterinary Medicine, Brawijaya University, Malang, Indonesia.; ^6^ Department of Bacteriology II, National Institute of Infectious Diseases, Tokyo, Japan.

**Keywords:** *M. tuberculosis*, *eccB*_5_, ATPase, cloning, expression

## Abstract

*Mycobacterium tuberculosis* (*M. tuberculosis*) is the causative agent of tuberculosis in human. One of the major *M. tuberculosis* virulence factors is early secretory antigenic target of 6-kDa (ESAT-6), and EccB_5_ protein encoded by *eccB_5_* is one of its components. EccB_5_ protein is a transmembrane protein in ESX-5 system. The aim of this study is to explore the characteristics of wild-type EccB_5_ and its mutant form N426I. We expressed the EccB_5_ protein by cloning the mutant and wild-type *eccB_5_* gene in *Escherichia coli* (*E. coli*). We compared the protein structure of wild type and mutant form of EccB_5_ and found changes in structure around Asn426 (loop structure) in wild type and around Ile426 (β-strand) in the mutant. The truncated recombinant protein of EccB_5_ was successfully cloned and expressed using plasmid pCold I in *E. coli* DH5α and *E. coli* strain Rosetta-gami B (DE3) and purified as a 38.6 kDa protein by using the affinity column. There was no detectable adenosine triphosphatase activity in truncated forms of EccB_5_ and its mutant. In conclusion, our study reveals successful cloning and protein expression of truncated form of eccB5 gene of *M. tuberculosis*. EccB_5_ protein in ESX-5 system may be an important membrane component involved in the transport machinery of type VII secretion system, which is essential for growth and virulence.

## Introduction

Tuberculosis (TB) is one of the major global public health problems. According to World Health Organization (WHO), Indonesia is regarded as a high TB burden country and holds the second highest position in the number of TB cases in the world. Incidence of TB cases in Indonesia is estimated to be about 1.02 million per year.^1,2^ WHO global TB report indicates that in 2017, Indonesia is still a high TB burden country, lies in third position for incident TB cases, after China.^3^

TB is an infectious disease in human caused by *Mycobacterium tuberculosis* and zoonotic TB is caused by *M. bovis*.^1,4–6^ *M. tuberculosis* is a pathogenic bacterial species of the mycobacteria genus and has many virulence factors and proteins secreted via type VII secretion systems (T7SSs) which are well documented.^7–9^ The T7SSs have five systems: ESX-1, ESX-2, ESX-3, ESX-4, and ESX-5. These secretion systems are important for virulence of mycobacteria, especially the EXS-5 system (Rv1782 - Rv1798) is only present in slow-growing mycobacteria.^10,11^ ESX-5 system has four core component (EccB_5_, EccC_5_, EccD_5_, and EccE_5_) the estimated size is about ∼1500 kDa.^12^ Satta et al., reported that mutation in the *eccB_5_* gene was observed in the virulence gene from isolates found during isoniazid-resistant outbreak in London.^13^ The single-nucleotide polymorphism (SNP) was detected in *eccB_5_* gene located at position 2017898, numbers annotated from whole-genome database of *M. tuberculosis,* accession number: PRJEB13764, which resulted in the reduction of colony-forming unit (CFU) count.^13^ We have found in our previous study that SNP in the *eccB_5_* gene of isolates from Indonesia in the open reading frame region at 1277th nucleotide change in A/T.^14^

The SNPs are the most common form of genetic variation, and the unique diversity is also observed in several genes. The SNPs could have the important effect on the phenotype of mycobacteria, which could influence the pathogenesis of *M. tuberculosis* infection, molecular epidemiology, demographic variations, phylogenetic markers, drug resistance, and gene functions.^15^

In this study, we focused on the characteristics of EccB_5_ of both wild type and mutant (1277 A/T) form of *eccB_5_* of *M*. *tuberculosis* by expressing the gene and purifying the recombinant protein. EccB_5_ has high homology to EccB_1_, which has one or more motif for adenosine triphosphatase (ATPase) activity, so we performed the docking experiment with the Swiss model found in the database and measured the ATPase activity of purified truncated EccB_5_ and its mutant.

## Materials and Methods

### Plasmid and bacterial strain

In this study, we used pCold I (Takara^®^, Shiga, Japan) as an expression vector and *Escherichia coli* (*E. coli*) strain DH5α as a bacterial cloning host. We used *E. coli* strain Rosetta-gami B (DE3) as an expression host. *E. coli* strain DH5α and Rosetta-gami B (DE3) were cultured for preparing the competent cells according to the standard protocol.^16^ *E. coli* strain DH5α were cultured in Luria-Bertani (LB) media containing antibiotic ampicillin 100 μg/mL. The *E. coli* Rosetta-gami B (DE3) were grown in LB medium containing tetracycline (12.5 μg/mL), chloramphenicol (34 μg/mL), kanamycin (15 μg/mL), and ampicillin (100 μg/mL).

### Protein prediction and determination of transmembrane protein

The protein structure prediction and homology was carried out using the Swiss Model.^17,18^ The protein model of EccB_5_ was built using 3x3n for template.^19^ Transmembrane helices proteins region prediction was used to check and confirm the protein structural details.^20,21^

### Construction of plasmid for expression of EccB_5_

The *eccB_5_* gene was constructed using *M. tuberculosis* gene and EccB_5_ protein was prepared in *E. coli* DH5α using the pCold I plasmid. Primer F EccB_5_ 418 Eco (5′-ATA TGA ATT CGT GGG TAT CCC GGG TGC G-3′) and R- EccB_5_ Xba (5′-TTA ATC TAG ATT TCG GTA CCA CCA ACT CTG-3′) used to amplify the truncated *eccB_5_* gene. The polymerase chain reaction (PCR) was performed by PrimeSTAR MAX DNA polymerase (Takara) using PCR thermal cycler according to the following set condition: predenaturation at 94°C for 2 min, denaturation at 98°C for 10 sec, annealing at 55°C for 5 sec, extension at 72°C for 5 sec, and total number of cycles are 20. Subsequently, PCR product was purified according to the protocol of DNA Purification Kit (Monarch^®^; New England Biolabs Japan Inc., Tokyo, Japan). The PCR product was subsequently ligated to the expression vector and the pCold I W 418 and pCold I M 418 were obtained. Sequencing was performed for confirmation of the DNA target sequences.

### Expression of recombinant protein of EccB_5_ in *E. coli* strain Rosetta-gami B (DE3)

The cloned plasmids were transformed into *E. coli* Rosetta-gami B (DE3). The single colony was picked and inoculated in 1.5 mL of LB broth containing appropriate antibiotics and incubated at 37°C for 24 h with shaking. Five hundred microliter of the bacterial cultures were transferred into 50 mL of LB broth containing adequate antibiotics and incubated at 37°C. After reaching the OD_600_ = 0.6, cells were induced by 1.0 mM isopropyl β-D-1-thiogalactopyranoside and then cultured at 15°C for 16 h for expression.

### Extraction and purification of recombinant protein

*E. coli* cells were harvested, and the wet weight was determined. Cells were extracted using Bug Buster^®^ (Merck, Tokyo, Japan) according to the manufacturer's protocol. The crude protein was purified using Talon^®^ metal resin **(**Clontech Laboratories Inc., CA). The extracted protein was dialyzed against 10 mM Tris HCl, pH 7.5.

### Sodium dodecyl sulfate-polyacrylamide gel electrophoresis and western blot

In brief, 4 μL of extracted protein samples were loaded on the wells of 12.5% sodium dodecyl sulfate-polyacrylamide gel electrophoresis (SDS-PAGE). The separated protein on SDS-PAGE was stained using Coomassie brilliant blue (CBB). Anti-His antibodies (primary antibody, Novagen^®^; EMD Chemical, Inc., Germany) and horse radish peroxidase-linked anti-mouse IgG (secondary antibody; Cell Signaling Technology, Inc.) were used for detection in a western blot (WB) analysis.

### ATPase activity

The ATPase activity was measured using the ATPase assay kit (Sigma-Aldrich), according to the protocol of Sigma-Aldrich. In brief, 10 μL of the concentrated samples were dispensed into microplate. Ten microliter of 4 mM ATP was added in 20 μL assay buffer and incubated at room temperature for 30 min. Two hundred microliter of the reagent was added to each well and was incubated for 30 min at room temperature. Readings at 600–650 nm was taken with enzyme-linked immunosorbent assay plate reader (Vmax).

### Molecular modeling of EccB_5_

Homology modeling was performed using the SWISS model. Modeling of the EccB_5_ hexamer was performed using SymmDock.^22^ The model with the highest geometric shape complementarity scores were chosen as the model of EccB_5_. Molecular operating environment (MOE) was used as the platform for the docking simulation with ATP.

## Results

### Prediction of protein structure of EccB_5_

**FIG. 1.**
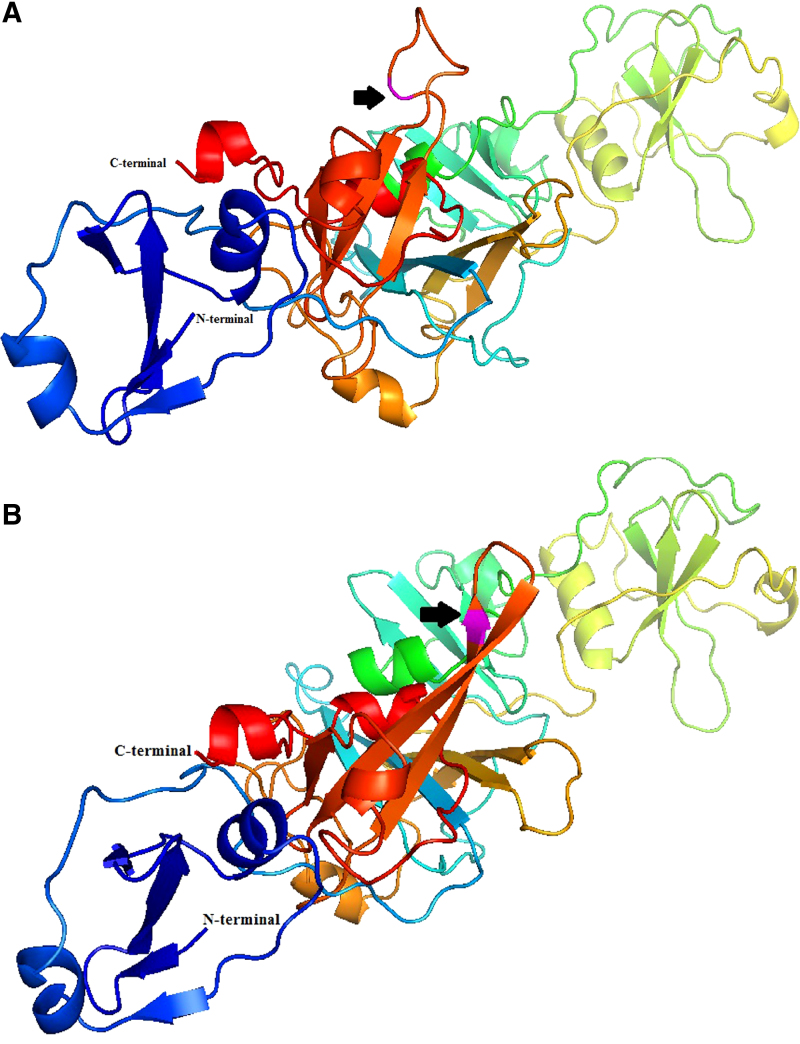

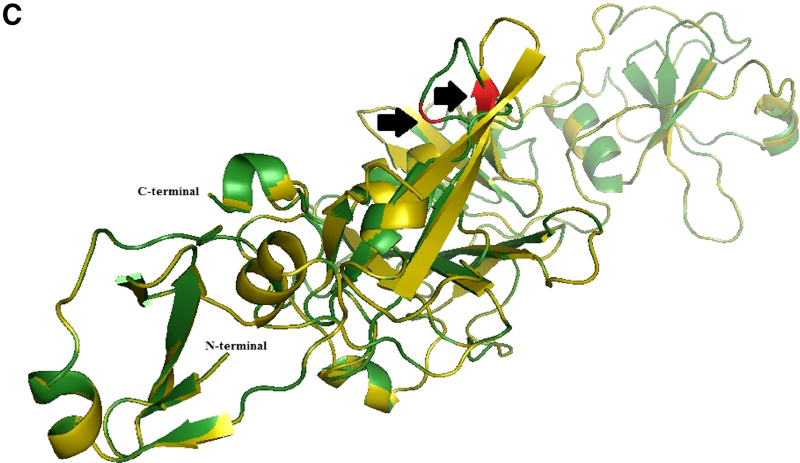
The predicted structure of EccB_5_ protein model. **(A)** Protein prediction model of EccB_5_, where of amino acid residue Asn426 is shown as a cartoon model (magenta). **(B)** Protein prediction model of mutated EccB_5_, amino acid residue Ile426 is shown as a cartoon model representation in magenta color. **(C)** Superimposition of wild-type and mutant EccB_5_. The β-strand structure for amino acid residue of Ile426 (mutant) in shown in red, which is in contrast to loop structure displayed in red for amino acid residue of Asn426 (wild type) (prediction models were prepared using PyMol software, Schrödinger). Arrows indicate the position of amino acid where SNP in the EccB_5_ occurred.

The prediction protein structure of EccB_5_ full form were explored by using PyMol software, the Molecular Graphic System, by Schrödinger, to locate mutation A1277T using 3x3n as template X-ray, 2.0 Å (Fig. 1). The secondary structure of wild-type EccB_5_ shows amino acid residues of Asn426 as a cartoon model in magenta (Fig. 1A). While amino acid residues of mutant EccB_5_ Ile426 in cartoon model (β-strand structure) are as shown in magenta (Fig. 1B), the superimposed structure of EccB_5_ protein wild type and mutant consists of α-helical structure (α1- α 10) and β-strand structure (β1–β17) are as shown in Figure 1C. Analysis of root mean square deviation between mutant and wild type is 0.015 Å, these indicate that both proteins have similar structure.

In this study, we tried to express recombinant EccB_5_ protein in *E. coli* strain Rosetta-gami B (DE3) in full form, but the results showed that the expression was negligible (data not shown). So, to increase the amount of protein, the nucleotide sequences at the N-terminal transmembrane region were deleted from the full form. Analyses showed that the transmembrane region of *eccB_5_* gene was at position 54–76 of amino acid residues.

In this study, the nucleotide sequences (1–417) were deleted and primer was designed to include the gene starting from 418 of nucleotide sequences to the stop codon. The molecular weight of truncated recombinant protein of EccB_5_ (rEccB_5_) was estimated to be ∼38.6 kDa.

### Expression of EccB_5_ in *E. coli* strain Rosetta-gami B (DE3)

**FIG. 2.**
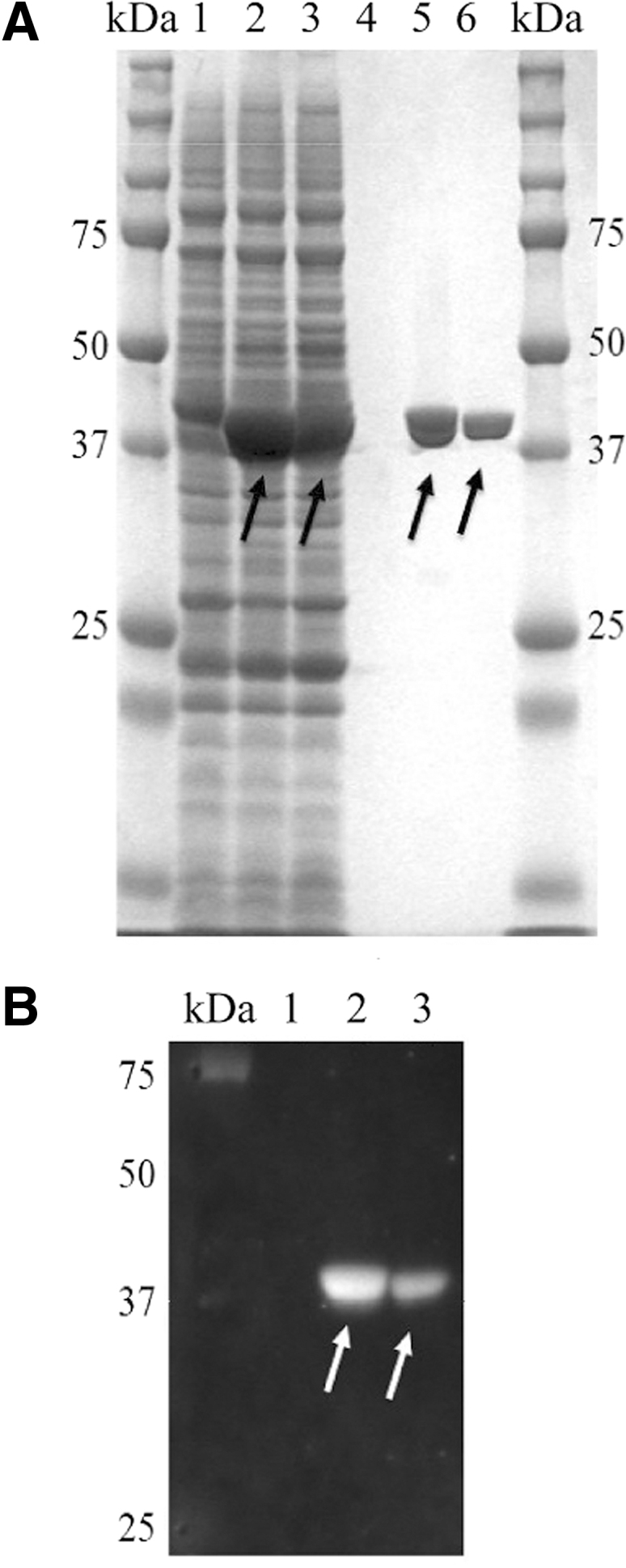
Expression of EccB_5_ in *Escherichia coli* strain Rosetta-gami B (DE3). **(A)** The protein was separated on 12.5% sodium dodecyl sulfate-polyacrylamide gel electrophoresis and stained using Coomassie brilliant blue. Molecular weight standards are indicated in kDa, lane 1: crude protein from *E. coli* strain Rosetta-gami B (DE3) carrying pCold I (vector control), lane 2: crude protein from *E. coli* expressing the truncated wild-type EccB_5_, lane 3: crude protein from *E. coli* expressing the mutant EccB_5_, lane 4: protein purified from vector control, lane 5: protein purified from truncated wild-type EccB_5_, and lane 6: protein purified from mutant EccB_5_. The black arrows indicate the expression of the EccB_5_ protein. **(B)** Western blot of protein EccB_5_ detected with His-Tag^®^ monoclonal antibody (1:2000) after purification of protein EccB_5_ with Talon^®^ metal resin. The white arrows indicate the position of the rEccB_5,_ lane 1: vector only, lane 2: truncated wild type, and lane 3: truncated mutant.

The expression of rEccB_5_ in *E. coli* strain Rosetta-gami B (DE3) is as shown in Figure 2A. The molecular weight of rEccB_5_ is 38.6 kDa and the bands are shown in black arrows. The target protein was extracted by using Bug Buster as shown in CBB staining (lane 1, 2, 3). Extracted protein was purified by using Talon metal resin. The SDS-PAGE of the purified protein after dialysis in 10 mμ Tris Hcl pH75 is shown as a distinct band in CBB stain (lane 5, 6, black arrows). It is clear that the protein of interest was purified up to a certain level of purity. WB analysis of purified rEccB_5_ using anti-His-Tag antibodies is shown in Figure 2B. It is clear that rEccB_5_ of wild type and mutant was expressed in *E. coli* and detected by the antibody.

### ATPase activity

The purified protein was collected and ATPase activity was measured. There was no significant ATPase activity in both mutant and wild-type rEccB_5_ expressed in *E. coli* Rosetta-gami B (DE3) (data not shown). Predictable ATPase motif PX2NLXSARL is present at the N-terminal region of EccB1, so it is reasonable that the truncated EccB_5_ has no ATPase activity.^19^ The N-terminal of EccB_5_ may be playing a major role in ATPase activity, but the study needs further evaluation.

### Modeling of the EccB_5_ protein

**FIG. 3.**
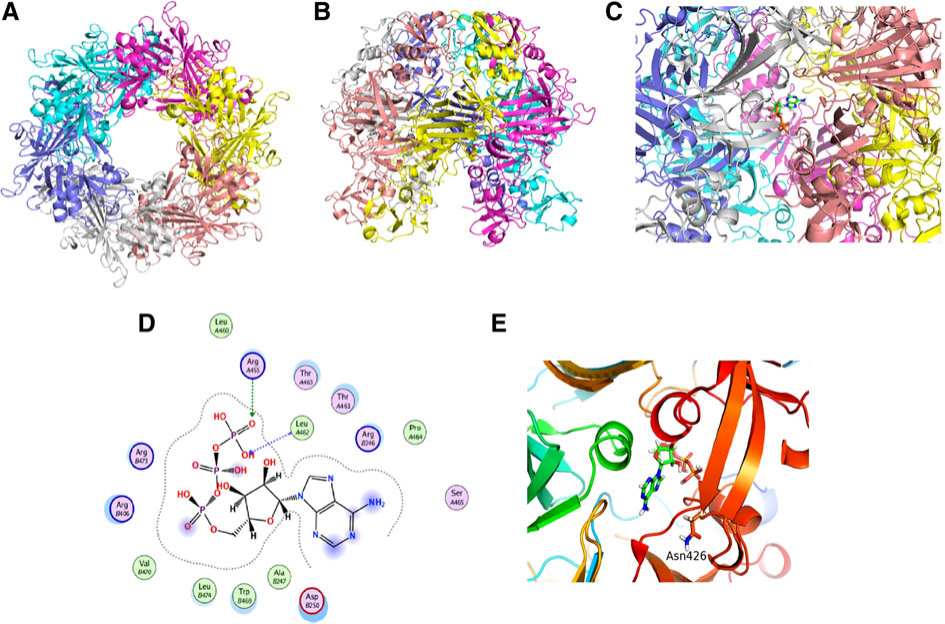
Putative models of EccB_5_ hexamer and ATP pocket. **(A)** Top view of the proposed sixfold rotational symmetric oligomer of EccB_5_. **(B)** Side view of the EccB_5_ hexamer. **(C)** The putative structure of ATP-binding site. ATP molecule is shown as a stick model. **(D)** The amino acid which were predicted to be localized within 4.5 Å from ATP molecule using molecular operating environment ligand interactions application. Green circles indicate the interaction between amino acid side chains with ATP and the blue circles show the interaction between amino acid backbone with ATP. **(E)** Enlargement of the binding pocket area of ATP. Asn426 region is shown as a stick model. ATP, adenosine triphosphate.

We analyzed the full length of EccB_5_ using SWISS model of EccB_5_ and the structure of the hexamer oligomer was predicted using SymmDock web server. Most ATPase functions as hexamers and so we hypothesized that the EccB_5_ forms a hexamer like that of EccB_1_.^19^ Figure 3A shows the predicted EccB_5_ hexamer as seen from the top, and the six monomers of EccB_5_ are indicated in different colors. In the middle of the hexameric oligomers is the small pore like gap, similar to the one seen in EccB_1_ predictable due to high homology to each other. The predicted structure of the EccB_5_ hexamer is as observed from the sides of the hexamer (Fig. 3B). This model was used to dock the ATP molecule into EccB_5_ hexamer. MOE was used as the platform for the simulation. The results of the simulation are as shown in Figure 3C. The ATP molecule is shown as a stick model and is located at the interface of EccB_5_. The ribbon model shows the location of ATP in EccB_5_ hexamer. The ligand interaction is predicted to be as shown in Figure 3D. The amino acids indicated are involved in the interaction with ATP. It is likely that Arg455 and Leu462 largely interact with ATP. Enlargement of the ribbon model at ATP interaction shows that Asn426 is adjacent to ATP-binding site (Fig. 3E). From the structural simulation, it seems likely that Asn426 is not directly involved in the interaction of EccB_5_ with ATP. The distance between the ATP and Asn426 is within 10 Å. In this simulation, neither water molecules nor metallic ions are included, so we cannot rule out the possibility that Asn426 is not directly involved in the interaction with ATP. Further experimentation with the crystallization of EccB_5_ with ATP may reveal the exact involvement of Asn426 in ATP binding.

## Discussion

TB is an infectious disease caused by *M. tuberculosis*. *M. tuberculosis* is member of mycobacteria tuberculosis complex and is included in the slow growing mycobacteria group. Slow-growing mycobacteria have one of the unique secretion systems, namely the ESX-5 system.^7,8,23^ ESX-5 system consists of 16 coding genes, one of them is *eccB_5_* gene.^9,24^

In previous study, the SNP in locus 1277 T/A of *eccB_5_* nucleotide sequences, which is known as nonsynonymous SNP (nsSNP), was found, and single amino acid change at position 426 from Aspartic acid residue to Isoleucine was observed.^14^ However, the function of the protein is not clear, so we expressed the recombinant *eccB_5_* gene and analyzed the wild-type and the mutated N426I EccB_5_ protein.

To know the structural differences of the EccB_5_ protein, prediction of the 3D protein models was done based on the database obtained from Swiss model and compared the structure of mutant and wild type using 3x3n template.^19^ The mutated region showed β-strand structure, while the wild type showed the loop structure prediction. These changes may be due to the changes in amino acid residues Asn426, which has hydrophilic side chain to Ile426, which has hydrophobic side groups. The structure of the hexamer oligomer was predicted using SymmDock web server as seen in Figure 3. This model was used to dock the ATP molecule into EccB_5_ hexamer. MOE was used as the platform for the simulation. The results of the simulation are as shown in Figure 3C. The ATP molecule is located at the interface of adjacent EccB_5_. Amino acids Arg455 and Leu462 have stronger interaction with ATP. Enlargement of the ribbon model at ATP interaction, shows Asn426 adjacent to ATP-binding site. The distance between the ATP and Asn426 is within 10 Å. In this simulation, neither water molecules nor metallic ions are included, so we cannot rule out the possibility that Asn426 is not directly involved in the interaction with ATP. Further experimentation with the crystallization of EccB_5_ with ATP may reveal the exact involvement of Asn426 in ATP binding.

Although we tried to express the full forms of *eccB_5_* gene, the protein expression was negligible. The result is similar to that obtained by Beckham et al.^25^ EccB_5_ expression is low probably due to the low stability of a single protein in the ESX-5 complex system. To overexpress the protein, we deleted the N-terminal transmembrane helices protein region of 417 nucleotide sequences. There are reports that the presence of the transmembrane protein results in extremely low level of the expressed protein.^26^ The truncated recombinant protein of wild-type and mutant EccB_5_ was successfully expressed in *E. coli* Rosetta-gami B (DE3). In addition, the growth of recombinant bacteria between *E. coli* expressing mutant and wild type was no different. The growth of *E. coli* expressing wild-type EccB_5_ was higher than those expressing the mutant EccB_5_. Interestingly, the yield of the mutated EccB_5_ was lower compared to the wild-type EccB_5_ as observed from the bands of the CBB stain. Purification does not alter the amount of protein obtained. Amino acid residues of Asn are often involved in active binding sites of the protein, probably due to their polar nature. On the contrary, amino acid residue of Ile is nonreactive and infrequently involved in direct protein function, but sometimes involved in substrate recognition due to the hydrophobic nature of the side chain.^27^ SNP of *eccB_5_* gene was also found in different locations of T/T, and reduced CFU count was observed in *M. tuberculosis*.^13^ The *eccB_5_* gene on the ESX-5 system in *M. tuberculosis* has important functions, such as viability, nutritional uptake, and cell wall integrity.^28^ Di Luca et al., also described that deletion of *eccB_5_-eccC_5_* gene affected bacterial growth of *Mycobacterium marinum*.^28^

In this study, we measured the ATPase activity, but there was no significant ATPase activity in both wild-type and mutant EccB_5_. In a previous study, it was shown that EccB_1_ is a periplasmic ATPase, and EccB_5_ is highly homologous to EccB_1_ in ESX system.^19^ Our EccB_5_ is expressed as a truncated form, so it may be possible that the ATPase function is lost when the N-terminal motif PX2NLXSARL is deleted. According to Zhang et al., ATPase motif PX2NLXSARL at N-terminal position has direct involvement in ATPase activity.^19^

ATPase is one of the important enzymes in bacteria, such as for driving substrates across the inner membrane using ATP dephosphorylation activity.^19,29^ EccB_5_ hexamer may function as a part of a transport channel of the huge type VII secretion membrane system. Houben et al., reported that EccB_5_ located at the cell envelope of *M. marinum* forms a large membrane complex along with EccC_5_, EccD_5_, and EccE_5_, and so it is likely that EccB_5_ of *M. tuberculosis* may be involved in the formation of channel spanning the plasma and mycobacterial membrane.^12^ Also as such, it may have an important role as virulence factor in *M. tuberculosis* and may be a possible target for drug design.

## Abbreviations Used

ATP: adenosine triphosphate
ATPase: adenosine triphosphatase
CBB: Coomassie brilliant blue
CFU: colony-forming unit
DNA: deoxyribonucleic acid
ESAT-6: early secretory antigenic target of 6-kDa
ESX: ESAT-6 secretion system
IPTG: isopropyl β-D-1-thiogalactopyranoside
ITD: Institute of Tropical Diseases
LB: Luria-Bertani
MOE: molecular operating environment
nsSNP: nonsynonymous SNP
PCR: polymerase chain reaction
rEccB_5_: recombinant protein of EccB_5_
RMS: root mean square
SDS-PAGE: sodium dodecyl sulfate-polyacrylamide gel electrophoresis
SNP: single-nucleotide polymorphism
T7SS: type VII secretion system
TB: tuberculosis
WB: western blot
WHO: World Health Organization
